# Psoriasis: an emerging risk factor for ischemic stroke?

**DOI:** 10.3389/fneur.2025.1599978

**Published:** 2025-06-13

**Authors:** Piotr Olejnik, Kaja Kasarełło, Renata Podkowińska-Polak, Aleksandra Golenia

**Affiliations:** ^1^Department of Neurology, Medical University of Warsaw, Warsaw, Poland; ^2^Chair and Department of Experimental and Clinical Physiology, Laboratory of Centre for Preclinical Research, Medical University of Warsaw, Warsaw, Poland; ^3^Department of Pathology, Medical University of Warsaw, Warsaw, Poland

**Keywords:** acute ischemic stroke, psoriasis, systemic inflammation, autoimmune erythematous-squamous disease, traditional cardiovascular risk factors

## Abstract

In 2020 nearly 12 million people worldwide suffered a stroke, and acute ischemic stroke (AIS) is the most frequent stroke subtype, accounting for approximately 65% of total stroke incidence. Therefore, primary prevention, including non-traditional risk factors, should be recognized as a major public health priority. Research has shown that autoimmune diseases associated with chronic systemic inflammation, such as psoriasis, are commonly linked to AIS incidence. Psoriasis is a chronic autoimmune erythematous-squamous disease that primarily affects the skin, nails, and joints. Psoriasis is known to be a systemic inflammatory condition affecting multiple organs. Patients with psoriasis are at a higher risk of stroke than the general population, and a more severe disease course can increase this risk by up to 44%. One possible explanation for this phenomenon is that chronic systemic inflammation is associated with endothelial dysfunction and atherosclerotic plaque development. On the other hand, patients with psoriasis have an increased prevalence of traditional cardiovascular risk factors, including metabolic syndrome. This narrative review synthesizes the scientific literature to provide a comprehensive overview of the current understanding of the association between psoriasis and AIS.

## Introduction

Stroke is the second leading cause of death and disability worldwide (1). In 2020, nearly 12 million people worldwide suffered a stroke, and acute ischemic stroke (AIS) is considered the most common subtype of stroke, accounting for approximately 65% of the total stroke incidence (2). Cheng et al. estimate that the global incidence of stroke will have exceeded 21 million cases by the year 2050 (3). Therefore, primary prevention, which includes not only the management and control of traditional cardiovascular risk factors such as hypertension, diabetes mellitus, dyslipidemia or atrial fibrillation but also lifestyle modifications, should be prioritized in public health (4). Inflammatory and infectious diseases have also long been considered potential risk factors for AIS, but the exact causal relationship remains uncertain (5). Recent studies indicate that autoimmune conditions associated with chronic systemic inflammation are often linked to AIS. Among these, rheumatoid arthritis appears to have the strongest association, yet there is also emerging evidence for the involvement of psoriasis in AIS pathophysiology (6).

Psoriasis is a chronic autoimmune erythematous-squamous disorder defined by proliferative changes predominantly affecting the skin, nails, and joints (psoriatic arthritis) (7–10). The prevalence of psoriasis varies from country to country, e.g., it affects only 0.05% of the general population in Taiwan, which makes it the country with the lowest psoriasis prevalence in the world (11). On the other hand, the prevalence of psoriasis in the United States has been estimated to be as high as 3%, making it one of the most common immune-mediated disorders there (12). Nonetheless, it is a common condition worldwide, affecting people of all ages (7) with a higher prevalence in adults, although it can also occur in children (11). Psoriasis affects both women and men, but in women, the onset typically occurs approximately 10 years earlier than in men, and is more common in patients with a positive family history of the disease (9). The pathophysiology of psoriasis has been linked to genetic susceptibility, in particular the presence of the HLA-C*06:02 risk allele, but also to environmental factors such as tobacco smoking, alcohol consumption, diet, obesity, low physical activity, streptococcal infections or stressful life events (7, 13). However, the exact etiology of psoriasis is complex and remains unclear (14).

For many years, psoriasis was considered a skin disease primarily treated with topical medications and, if necessary, phototherapy. It is now well known that psoriasis is a systemic inflammatory condition with multi-organ involvement (15). This state of chronic low-grade inflammation, usually subclinical, may be associated with comorbidities such as obesity, diabetes mellitus, non-alcoholic fatty liver disease, and cardiovascular disorders (15–17). For instance, according to a systematic review conducted by Correa et al., five out of eight studies included in their analysis indicated that psoriasis increased the risk of myocardial infarction (18). In addition to atherosclerotic diseases, psoriasis might be associated with increased risk of heart failure (19), also in in younger populations (20). According to a cross-sectional study conducted by Eckembrecher et al., among 2,485 hospitalizations of psoriasis patients, 13.7% had comorbid cardiovascular disease (21). Moreover, these individuals tended to have significantly longer hospital stays and generated higher medical costs compared to those without cardiovascular disease (21). Additionally, the risk of developing cardiovascular disorders in psoriatic patients increases with the severity of the underlying disease (17). Notably, the treatment of psoriasis can also modulate the cardiovascular risk profile, with tumor necrosis factor (TNF)-*α* inhibitors and methotrexate demonstrating beneficial effects (22).

**Figure 1:**
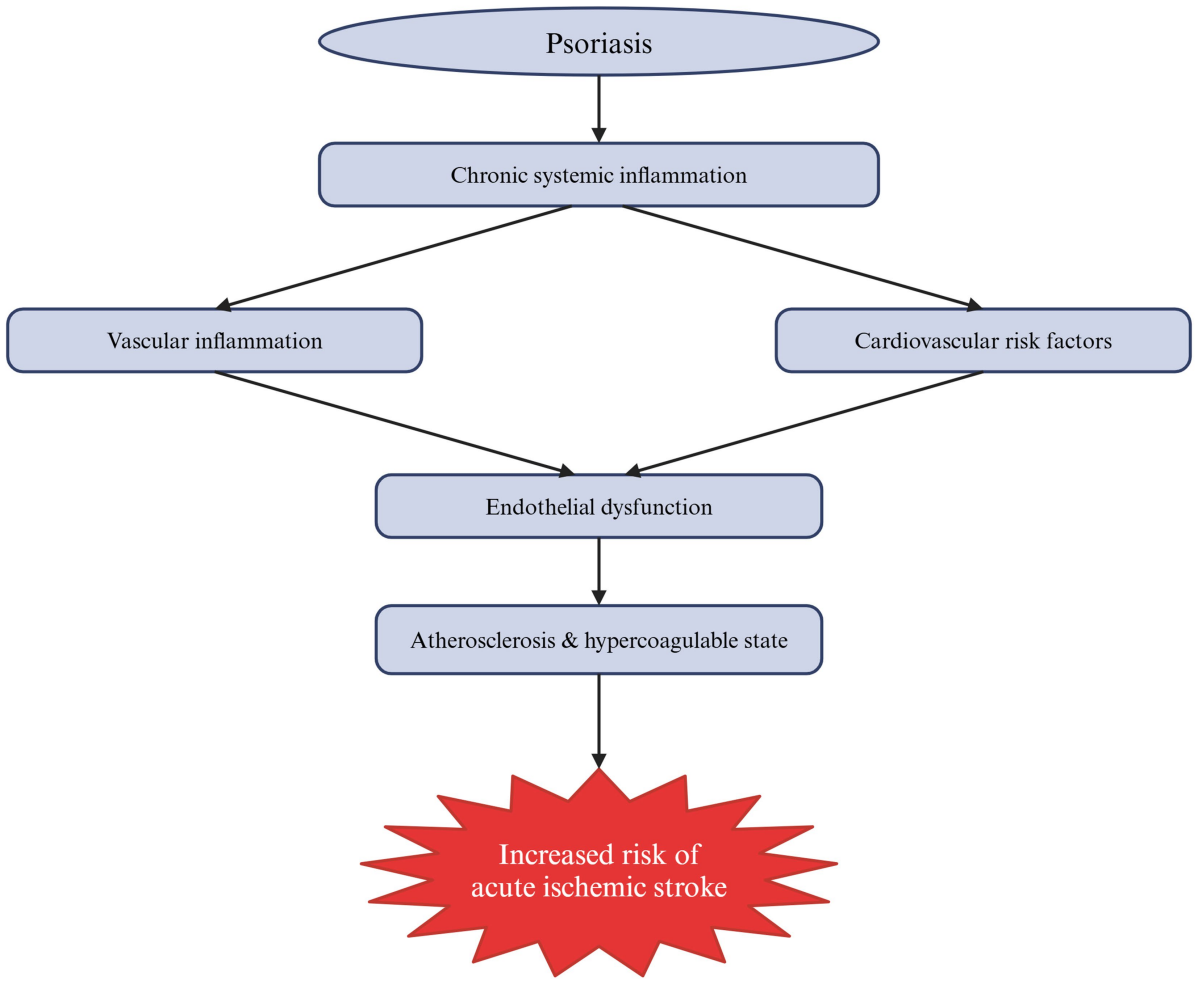
Schematic summary of the mechanisms by which psoriasis contributes to the increased risk of acute ischemic stroke (AIS). As a systemic autoimmune disease, psoriasis is associated with chronic low-grade inflammation that directly affects blood vessels, causing their inflammation, and indirectly influences cardiovascular risk factors. Collectively, these mechanisms lead to endothelial dysfunction, atherosclerosis and a hypercoagulable state, ultimately increasing the risk of AIS.

Also, patients with psoriasis are at a higher risk of stroke than the general population, and a more severe course of the disease may increase this risk by 44% (23). A large Danish cohort study based on nationwide registries found that psoriasis is linked to an increased risk of atrial fibrillation (AF), one of the major AIS risk factors in the elderly, as well as to a severity-dependent increase in AIS risk (24). This relationship is complex and not fully understood. Nonetheless, psoriasis is associated with AIS not only through risk factors that are more common in patients with psoriasis but also through shared inflammatory pathways (16, 23). Moreover, endothelial dysfunction might represent a key mechanistic link between chronic systemic inflammation and the elevated cardiovascular risk in patients with psoriasis (25). Finally, in a study by Gelfand et al., the increased risk of stroke was present regardless of the treatment used, whether it potentially increased it, like oral retinoids, or theoretically reduced it, such as methotrexate (26). Figure 1 summarizes the multifaceted connection between psoriasis and AIS.

This narrative review aims to summarize the scientific literature and provide a comprehensive overview of the current understanding of the link between psoriasis and AIS, highlighting the potential role of the inflammatory pathomechanism underlying psoriasis in contributing to AIS incidence. It also explores psoriasis connection to traditional AIS risk factors.

## Methodology

A comprehensive literature search was conducted using the PubMed and Google Scholar databases, covering studies from their inception to 5 February 2025. Both experimental and clinical studies were analyzed. A review of the current literature on the relationship between psoriasis and AIS identified key areas to be explored in this review. The pathophysiology of psoriasis and its role in systemic inflammation is discussed, as is the contribution of inflammation as a risk factor for AIS. Also, the analysis extends beyond inflammatory pathways to examine the role of psoriasis-associated comorbidities in stroke development. Titles and abstracts were searched for key terms such as ‘psoriasis’, ‘systemic inflammation’, and ‘acute ischemic stroke’. However, the major limitation of this review is the predominance of observational and cross-sectional studies, which by design, are unable to establish temporal relationships or causality. These studies are prone to a variety of biases, including selection bias. Moreover, the lack of randomized or longitudinal data limits causal inference and underscores the need for more rigorous prospective studies to validate the associations described in this paper. To ensure comprehensive coverage, relevant references from identified articles were also manually reviewed. However, articles written in languages other than English and papers not published as full scientific papers, such as conference abstracts, were excluded from our search to ensure the relevance of the review. The literature search was conducted independently by three authors (PO, KK, RPP) in January and February 2025.

## Pathophysiology of psoriasis

Psoriasis is a chronic systemic inflammatory disease that primarily affects the skin and involves uncontrolled epidermal hyperproliferation and differentiation of keratinocytes (27). Its pathogenesis involves dysregulation of the immune system. The pathogenesis of plaque psoriasis can be divided into an initial phase, which can occur spontaneously or might be potentially caused by trauma, drugs or infection, and a maintenance phase driven by the immune response via different T cell subsets (27, 28). The interleukin (IL)-23/IL-17 axis has been identified as the central cytokine network that drives disease (27). IL-17 binds to its receptor on keratinocytes and induces the production of keratinocyte antimicrobial peptides (AMPs). The most studied psoriasis-associated AMPs include LL-37, *β*-defensins, and S100 proteins (29). AMPs are typically overexpressed in psoriatic skin (28, 30), and they play a key role in the activation of plasmacytoid dendritic cells (pDCs), which promotes myeloid dendritic cells (mDCs) maturation (28, 30). Activated mDCs migrate to draining lymph nodes and secrete TNF-*α*, IL-12, and IL-23, leading to the proliferation of Th1 and Th17 cell subsets and secretion of inflammatory cytokines such as TNF-α, IL-17, IL-21, and IL-22 (28, 30). Th17 cytokines, namely IL-17, IL-21, and IL-22, activate keratinocyte proliferation in the epidermis; activated keratinocytes produce antimicrobial peptides, cytokines, and chemokines, thereby strengthening the IL-23/IL-17(A) axis (28, 30).

TNF-*α*, IL-17 and IL-23, are the key cytokines driving psoriasis, contributing to epidermal hyperproliferation and inappropriate keratinocytes differentiation, vascular angiogenesis and inflammatory cell infiltration (31–34). Epidermal hyperproliferation leads to acanthosis (thickening of other epidermal layers), hyperkeratosis (thickening of the stratum corneum), and accelerated cell turnover: keratinocytes retain their nuclei in the stratum corneum (parakeratosis) whereas normally they lose their nuclei in the stratum granulosum (35). This disruption of programmed keratinocyte differentiation results in a markedly thin or absent stratum granulosum (35). Cytokines (TNF-*α*, IL-17) stimulate the production of vascular endothelial growth factor (VEGF) and endothelial cells, leading to the proliferation of new capillaries (36, 37). VEGF levels are elevated in psoriatic plaques (36, 37). Inflammation results from the infiltration of neutrophils into the epidermis and superficial dermis and an infiltration of T lymphocytes into the dermis, with a predominance of CD8^+^ cells (29). IL-17A and IL-17F are two of several cytokines, capable of attracting neutrophils to the site of inflammation. LL-37, an AMP, is one of two well-studied T cell autoantigens in psoriasis (29). CD8^+^ T cells activated by LL-37 exhibit epidermotropism, recognize autoantigens, and secrete Th17 cytokines (38). Different pathomechanisms are associated with different psoriasis subtypes (38). While the TNFα–IL23–Th17 axis plays a central role in T cell-mediated plaque psoriasis, the innate immune system appears to play a more prominent role in the pustular variants of psoriasis (38). In guttate psoriasis, streptococcal superantigens are thought to stimulate the T cells expansion in the skin (39).

## Systemic inflammation in psoriasis

**Figure 2:**
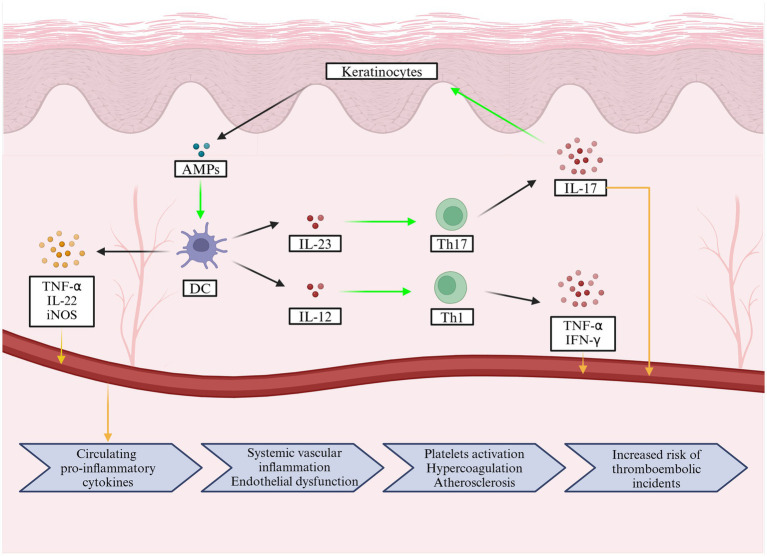
The pathophysiology of psoriasis involves cellular mechanisms in the skin that lead to the release of inflammatory mediators into the bloodstream, causing chronic systemic inflammation that can affect the entire body. In addition, a flowchart below illustrates the pathological changes caused by pro-inflammatory cytokines and how they increase the risk of acute ischemic stroke.

It is now well established that psoriasis is not merely a disease of a single organ; rather, inflammatory cytokines generated during its development and progression contribute to systemic inflammation affecting the entire body (15). Although reported results vary, it is generally assumed that serum levels of proinflammatory cytokines, such as TNF-*α*, IFN-*γ*, IL-6, IL-8, IL-12, IL-17A, IL-18 and IL-22, are elevated in patients with psoriasis and correlate with disease severity (40–43). Interestingly, Arican et al. reported no statistical difference in IL-17A levels between controls and patients with psoriasis (40), although this is a Th1- and Th17-mediated disease (44). In psoriasis, T cells are primarily stimulated by DCs, which are abundant in the skin. Activated T cells are responsible for the production of such cytokines as TNF-*α*, IFN-*γ*, IL-17, and IL-22 (44–46). Moreover, DCs themselves produce other cytokines, such as TNF-α and IL-20, as well as inducible nitric oxide synthase (iNOS), which is responsible for subsequent innate immune responses (44–46). All inflammatory mediators are produced locally in the skin lesions, but they are also released into the bloodstream, resulting in chronic systemic inflammation that may affect the entire body (illustrated in Figure 2) (44). Dowlatshahi et al. investigated serum levels of inflammatory markers in patients with psoriasis. The meta-analysis revealed that five out of the six investigated pro-inflammatory markers studied were elevated compared to healthy controls (IL-6, C-reactive protein (CRP), intracellular adhesion molecule (ICAM)-1, E-selectin, TNF-*α*). No differences were found for the pro-inflammatory IL-1β and the anti-inflammatory IL-10 (47).

Many comorbidities have been identified in patients with psoriasis, resulting from systemic inflammation, which, may, in turn, impact psoriasis progression (44). Hypertension, cardiovascular disease, metabolic syndrome, fatty liver disease, diabetes mellitus, inflammatory bowel disease, psoriatic arthritis and others, most of which are Th17 and/or TNF-*α* dependent, have been reported in patients with psoriasis (15, 48, 49). Pro-inflammatory cytokines are known to be involved in the pathophysiological mechanism of the above diseases, indicating the contribution of systemic inflammation to the dysfunction of many organs (15, 49, 50).

## Vascular inflammation

Arterial inflammation is a recognized feature of psoriasis (49). The formation of initial non-calcifying atherosclerotic plaque and endothelial inflammation has been observed, with the involvement of IL-6, IL-17 and TNF-*α*, whose levels are elevated in psoriasis, being postulated (49, 50). Cellular contribution involves T cells (Th1 and Th17) and neutrophils, and the influence of platelets has been observed (51). There is growing evidence highlighting the importance of Th17-dependent responses. Th17 cells present in atherosclerotic coronary arteries isolated from human patients produce IL-17 and IFN-*γ*, both of which are responsible for inducing the immune response in the vessel smooth muscles and further neutrophil infiltration (51). The involvement of Th17 cells in atherosclerotic plaque development has also been confirmed in ApoE−/− mice (52). Moreover, the number of Th17 cells was even increased in late-stage plaques. The effect was suppressed by the administration of IL-17 neutralizing antibodies to the mice (52). IL-17 neutralizing antibodies also increased plaque stability in ApoE−/− mice (53). Innate immune cells such as neutrophils, monocytes and macrophages are also found both in psoriatic and atherosclerotic plaques, leading to overall disease exacerbation (54). Similarly, platelet engagement as a source of immune mediators is observed in both diseases (54). Moreover, increased platelet aggregation has been observed in psoriasis patients, which may contribute to cardiovascular disorders (55). Proinflammatory cytokines, IL-6 and TNF-*α*, which are elevated in patients with psoriasis, are also involved in the stimulation of prothrombotic factors that may result in thrombosis (44).

As elevated levels of proinflammatory cytokines in systemic inflammation lead to blood–brain barrier (BBB) opening and subsequent immune infiltration into the central nervous system (CNS) (56, 57), this mechanism may also occur in CNS vasculature.

## Psoriatic systemic inflammation as a risk factor for acute ischemic stroke

Chronic infections as well as systemic autoimmune inflammatory conditions have been linked to AIS (5, 58). Low-grade systemic inflammation, which is one of the characteristic features of psoriasis (15), is widely recognized as a triggering factor for atherosclerosis progression and plaque destabilization (59). Patients with psoriasis exhibit elevated levels of CRP compared to healthy individuals (47, 60). Moreover, patients with severe disease course, as indicated by the Psoriasis Area Severity Index (PASI) > 10, showed significantly higher levels of CRP than those with mild disease (60). Finally, CRP levels were significantly higher in patients with psoriasis and metabolic syndrome compared to those without (60). CRP is known to be associated with an increased risk of stroke incidence (61), which highlights the link between systemic inflammation and stroke. Moreover, IL-17A, the key pathophysiological driver of psoriasis (62), also contributes to the pathological process of AIS (63). Although the role of Th17 cells and IL-17 in the pathogenesis of AIS is not fully understood, research suggests that they may be involved in the development of atherosclerosis (63, 64). Psoriasis-related inflammation is also associated with endothelial activation, as evidenced by significantly increased levels of sICAM-1 in patients with psoriasis compared to controls (65). Additionally, there is evidence of platelet activation in these patients, as demonstrated by significantly elevated concentrations of sP-selectin (following adjustments for age and gender) compared to controls (65). Ultimately, all of the above link psoriasis to an altered coagulation profile, characterized by an increased likelihood of blood clot formation, as proven by thromboelastography (65). In a study by Zhao et al. over 55% of 267 patients with psoriasis had elevated fibrinogen levels (66). However, Marongiu et al. found no significant differences in fibrinogen levels between psoriasis patients and controls (67). Nonetheless, scanning electron microscopy shows fibrin clots in psoriasis patients to be denser than in healthy controls, with a larger fibrin fiber diameter associated with the disease (65). Additionally, elevated fibrinogen levels are linked to an increased risk of AIS (68).

## Psoriasis and stroke association—beyond chronic inflammation

Patients with psoriasis might face complications directly related to both chronic systemic inflammation and those associated with elevated higher prevalence of metabolic syndrome (69). Metabolic syndrome is an umbrella term that can be described as a concomitance of traditional cardiovascular risk factors, including obesity, insulin resistance, atherogenic dyslipidemia and hypertension (70). According to a recent study by Almenara-Blasco et al., obesity, dyslipidemia, and diabetes mellitus are among the most common comorbidities in patients with psoriasis (71). These are major risk factors for AIS, which are also interrelated. For instance, central obesity, which is common in AIS patients, is significantly associated with diabetes mellitus (72). Thus, the metabolic syndrome is a self-perpetuating group of pathologies in which insulin resistance and chronic low-grade inflammation create a vicious cycle that exacerbates disease progression and increases the risk of developing diabetes mellitus and cardiovascular complications (73).

### Psoriasis, obesity and ischemic stroke

Obesity is a common risk factor for both psoriasis (74) and AIS (75). A recent meta-analysis by Wang et al. has revealed that 25% of patients with psoriasis worldwide also suffer from obesity, but this rate is notably higher in adults, at 35% compared with 18% in children and adolescents (74). Moreover, the comorbidity of obesity and psoriasis is more prevalent in women (38%) than in men (23%) (74). Obesity is associated with various pathological conditions due to the endocrine dysregulation of adipose tissue it induces (76). Obesity promotes the secretion of pro-inflammatory adipokines, including leptin, resistin, and lipocalin 2, which can contribute to chronic low-grade inflammation and cardiovascular complications (76). Adipokines may also be the key molecules linking psoriasis to the metabolic syndrome (77). However, the role of adipokines in the pathophysiology of psoriasis is unclear. The changes in adipokine levels in psoriasis and their potential roles in AIS pathophysiology are presented in Table 1. For example, adiponectin appears to have potentially beneficial effects on the course of psoriasis, as its levels were negatively correlated with IL-17 and IL-23, which are among the most important drivers of psoriasis (78). Kaushik et al. conducted a study and found significantly lower serum adiponectin levels in psoriasis patients compared to the controls (79). Even within the psoriasis group, adiponectin levels did not vary based on the presence of metabolic syndrome. Finally, there was a negative correlation between adiponectin levels and PASI, showing that decreased adiponectin levels are associated with a more severe course of psoriasis (79). Moreover, Chen et al. demonstrated that patients with AIS had significantly lower plasma adiponectin levels than those without ischemic cerebrovascular disease (80). Similarly, Efstathiou et al. indicated that reduced plasma adiponectin levels were independent predictors of an increased five-year mortality risk after a first AIS (81). Therefore, in general, adiponectin has protective effects on the cardiovascular system, as it exhibits both anti-inflammatory and antioxidative properties (82). On the contrary, leptin, visfatin and resistin appear to be positively correlated with psoriasis activity (78). Leptin is a pro-inflammatory agent that induces IL-1, IL-6 and TNF-*α* production (83). Therefore, the findings of the study by Xue et al. showing a positive correlation between serum leptin levels and PASI in overweight and obese male patients with psoriasis appear to be well-supported (84). Interestingly, a Mendelian randomization analysis by Dai et al. found no causal association between leptin levels and AIS (85). On the other hand, a study by Menon and Krishnan reported a significant increase in serum leptin levels in patients with AIS compared to the control group (86). Moreover, serum leptin levels were positively correlated with carotid intima-media thickness, suggesting a potential role of leptin in atherosclerosis (86). However, these findings must be interpreted with caution due to the small sample size of only 52 AIS patients, limiting the generalizability of the results (86). Additionally, another study suggested that elevated serum leptin and visfatin levels were significantly higher in 35 patients with AIS compared to controls (87). Visfatin levels are significantly higher in patients with psoriasis compared to controls and are positively correlated with PASI scores (88). Visfatin has regulatory properties in cell proliferation and apoptosis and has also been shown to induce TNF-*α*-induced chemokine secretion in human keratinocytes, linking it to psoriasis pathophysiology (78). According to a study by Yu et al., elevated levels of visfatin might be associated with an increased risk of AIS (89). Additionally, a meta-analysis by Agbaedeng et al. indicates that leptin, resistin and visfatin are associated with an increased risk of stroke, but not with new stroke events (90). Resistin exibits pro-inflammatory properties associated with its ability to induce IL-6 and TNF-*α* secretion. It also acts through the NF-κB pathway, a key regulator of inflammation (91). Moreover, as its name suggests, resistin influences insulin resistance and lipid metabolism, linking it to diabetes mellitus and dyslipidemia (91).

**Table 1:** Comparison of adipokines levels in psoriasis and their potential role in acute ischemic stroke (AIS).

Adipokine	Levels in psoriasis	Potential role in AIS
Adiponectin	Decreased in psoriasis compared to healthy controls; negatively correlated with disease severity	Generally protective effect for the cardiovascular system; reduced levels in AIS patients compared to controls; decreased adiponectin independently predicts increased 5-year mortality after first AIS
Leptin	Increased; positively correlated with disease severity	Pro-inflammatory effects (induces IL-1, IL-6, TNF-α production); studies show higher levels in AIS compared to controls
Resistin	Increased; positively correlated with disease severity	Pro-inflammatory effects (via IL-6, TNF-α, NF-κB pathway); influences insulin resistance and lipid metabolism
Visfatin	Increased; positively correlated with disease severity	Risk factor for AIS; involved in vital cellular functions, including cell proliferation and apoptosis

### Psoriasis, diabetes mellitus, dyslipidemia and ischemic stroke

Diabetes mellitus and atherosclerosis are closely related conditions, traditionally associated with an increased risk of AIS (92), with this risk being even higher in young patients with diabetes (93). Diabetes mellitus and psoriasis are also bidirectionally associated. Psoriasis is a well-established risk factor for the development of diabetes mellitus, while diabetes mellitus is associated with psoriasis exacerbation (94). Moreover, a meta-analysis by Armstrong et al. emphasized a stronger association between severe psoriasis and diabetes mellitus (95). Yang et al. identified potential mechanisms underlying the association between psoriasis and diabetes mellitus, emphasizing common signaling pathways such as Rap1, PI3K-Akt, and cGMP-PKG, along with key gene hubs such as SNRPN, GNAS, and IGF2 (96). Moreover, Brazzelli et al. demonstrated that fasting plasma insulin levels were significantly increased in patients with concomitant diabetes mellitus and psoriasis compared to those with diabetes or psoriasis alone and were significantly elevated in patients with psoriasis compared to controls (97). This is consistent with other studies suggesting that patients with psoriasis are more insulin resistant than healthy controls, which is thought to result from chronic systemic inflammation (98). Another possible explanation for insulin resistance in psoriasis could be its association with metabolic syndrome and elevated serum levels of resistin (91). Furthermore, according to a meta-analysis conducted by Untaaveesup et al., involving 8 studies with over 100,000 individuals, revealed 4 times increased risk of prevalent metabolic dysfunction-associated steatotic liver disease (MASLD) in patients with moderate-to-severe psoriasis (99). Hence, the co-occurrence of MASLD and diabetes mellitus might significantly contribute to insulin resistance in this population (100). What is more, biological treatment of psoriasis might also further increase the risk of MASLD (101), collectively creating a self-perpetuating cycle of metabolic dysfunction. Costanzo et al. present a case of a 73-year-old male patient with type 2 diabetes mellitus and treatment-resistant psoriasis, including a lack of response to adalimumab (102). Interestingly, their patient’s condition improved following treatment with semaglutide, suggesting the efficacy of glucagon-like peptide 1 (GLP-1) analogs in alleviating psoriasis symptoms (102). This effect might be associated with the inhibition of the NF-κB pathway by GLP-1 analogs, causing immunosuppression (103). Therefore, GLP-1 receptor agonists represent a promising treatment option for psoriasis patients with comorbid metabolic syndrome, which simultaneously might decrease the risk of stroke (104). Also, GLP-1/glucose-dependent insulinotropic polypeptide (GIP) receptor agonists might improve the course of MASLD (105, 106), which in turn can decrease the risk of diabetes mellitus, subsequently reducing the incidence of stroke (107). Furthermore, Brazzelli et al. also found that patients with psoriasis and those with concomitant diabetes and psoriasis exhibited significantly elevated levels of triglycerides, low-density lipoprotein-cholesterol (LDL-C) and homocysteine compared to patients with diabetes alone, further increasing their cardiovascular risk (97). These findings are supported by research by Nakhawa et al., who found a significant increase in total serum cholesterol, triglycerides, very low-density lipoprotein-cholesterol (VLDL-C) and high-density lipoprotein-cholesterol (HDL-C) in patients with psoriasis (108). However, this study found that LDL-C levels remained similar between patients with psoriasis and controls (108). Finally, recent studies emphasized the potential role of cutaneous microbiota in mediating the link between metabolic syndrome and psoriasis. Therefore, the emerging treatment strategies might be developed targeting these relationships (109).

### Psoriasis, hypertension, atrial fibrillation, and ischemic stroke

Finally, hypertension, considered one of the main risk factors for AIS and found in more than 80% of patients with AIS (110) is also more prevalent in patients with psoriasis compared to the general population (111, 112). A meta-analysis by Armstrong et al. showed the odds ratio for hypertension in patients with psoriasis to be 1.58 compared to controls (111). Furthermore, the odds ratio for hypertension is higher in patients with severe psoriasis (1.49) than in those with mild disease (1.30), suggesting an association between psoriasis severity and hypertension (111). Finally, psoriasis was found to be associated with uncontrolled hypertension. Nonetheless, this association was not statistically significant (113). The association between psoriasis and hypertension is thought to be related to common immunological pathways in both conditions (114). Karbach et al. conducted a study using a mouse model (K14-IL-17A^ind/+^model), in which conditional overexpression of IL-17A in keratinocytes caused psoriasis-like skin inflammation (115). The study found that the overproduction of IL-17A in the skin resulted in systemic vascular dysfunction, including hypertension. This suggests a causative link between psoriasis-induced systemic inflammation and vascular pathologies (115). Moreover, the IL-17 signaling pathway is thought to be involved in the pathophysiology of AF, as genes related to this signaling pathway are highly expressed in patients with AF (116). In addition to IL-17, TNF-*α*, another psoriasis-associated cytokine, has also been shown to be linked to the pathogenesis of AF, as demonstrated by animal studies (117). Furthermore, the activation of the TGF-*β* signaling pathway leads to decreased immunofluorescence of connexin-40 (117), one of the primary gap-junction proteins in the atrial myocardium, which is considered to be associated with AF (118). Therefore, given the involvement of inflammatory pathways and their impact on key cardiac proteins, the significantly elevated risk of AF in patients with psoriasis is not surprising (119). Also, psoriasis is associated with coronary atherosclerosis, a major contributor to coronary artery disease (CAD) (120). Recent evidence highlighted that AF is more prevalent in patients with CAD compared to age-matched adults without it, potentially due to the exacerbation of coronary atherosclerosis by AF through endothelial dysfunction and inflammation (121). Finally, according to a Korean nationwide population-based study by Rhee et al., patients with severe psoriasis not only had a higher incidence of AF, with an adjusted hazard ratio of 1.4, but also experienced more thromboembolic events, with an adjusted hazard ratio of 1.26 (122). This highlights the considerable cardiovascular risks associated with severe psoriasis.

## Therapeutic implications and future directions

The research consistently emphasizes the association between psoriasis and AIS (24). A more severe psoriasis course also correlates with a higher risk of stroke (24, 26, 122). Therefore, effective disease management seems to be crucial in mitigating these risks. On the other hand, it is important to note that some psoriasis treatments may also increase the risk of AIS (123). According to a large, nationwide population-based case–control study in Taiwan, treatment of psoriasis and/or psoriatic arthritis with cyclosporine, non-steroidal anti-inflammatory drugs (NSAIDs), or glucocorticoids was associated with an increased risk of hypertension (124). NSAIDs, including both cyclooxygenase 2-selective and nonselective medications, are known to be related to increased risk of cardiovascular events (125), whereas current glucocorticoids usage elevated the risk of 30-days mortality following AIS (126). Also, cyclosporine might increase the risk of AIS due to its hypertensive effects, though, to date evidence is insufficient to establish direct link between cyclosporine and AIS risk (127). Interestingly, cyclosporine was analyzed in AIS as an additional therapy to intravenous thrombolysis, however it did not significantly affect 30-day infarct volume compared with thrombolysis alone (128). Moreover, retinoids, including acitretin, one of the most commonly used drugs for the treatment of generalized pustular psoriasis (123), are typically associated with dyslipidemia (123, 129). Additionally, over 30% of patients treated with retinoids may experience abnormalities in their triglyceride levels (129), which elevates the risk of AIS by triggering atherosclerosis, thrombosis and increasing blood thickness (130). Ahlehoff et al. conducted a large study using a Danish nationwide cohort to assess the influence of systemic anti-inflammatory agents on the risk of cardiovascular events in patients with severe psoriasis. Interestingly, over a five-year follow-up period, methotrexate was associated with a reduced risk of cardiovascular death composite outcomes, myocardial infarction, and stroke compared with other therapies (131). Furthermore, treatment with TNF-*α* inhibitors was associated with a significant reduction in cardiovascular risk compared with other therapies, including the interleukin-12/23 inhibitor (i.e., ustekinumab) (131). A study by Wu et al. compared 9,148 patients with psoriasis treated with TNF-*α* inhibitors and 8,581 patients treated with methotrexate (132). The findings indicate that treatment with TNF-*α* inhibitors (i.e., adalimumab, etanercept, infliximab) reduced the risk of stroke and transient ischemic attack by almost 50%, compared to treatment with methotrexate (132). Furthermore, the treatment of psoriasis with TNF-*α* inhibitors may also have beneficial effects on the development of atherosclerosis and components of the metabolic syndrome, such as improving endothelial function, reducing the risk of diabetes mellitus, and improving insulin sensitivity (132). However, some studies have shown that TNF-α inhibitors are associated with an increase in body weight and body mass index in patients with psoriasis (123). In addition to TNF-α inhibitors, biological treatments for psoriasis include anti-IL-17 agents such as secukinumab, and no increased incidence of major cardiovascular events has been reported with these treatments (123). Moreover, given the association of IL-17 with AIS risk factors (115, 116), anti-IL-17 medications represent an interesting therapeutic option for the prevention of cardiovascular events. Thus, further research is needed to investigate this association (133).

In conclusion, when initializing systemic psoriasis treatment, it is crucial to consider cardiovascular safety, including baseline risk factors. Treatment with methotrexate and TNF-*α* inhibitors appears to be the most effective in reducing cardiovascular events in patients with psoriasis (134). Additionally, effective control and management of concomitant risk factors are crucial for optimal outcomes (23). Finally, newly developed treatments need to be carefully analyzed for their cardiovascular risk profile to ensure that they do not inadvertently increase the risk of these events, including AIS.

## Conclusion

Psoriasis is a common disease worldwide and has been recognized as a systemic inflammatory condition rather than solely a cutaneous disorder. Psoriasis shares pathophysiological pathways with AIS, including inflammatory mechanisms and a higher incidence of traditional cardiovascular risk factors in patients with psoriasis. As a result, both mild and severe psoriasis are recognized as independent risk factors for stroke (26). Moreover, the risk of stroke is influenced not only by the disease itself and its pathological processes but also by the medications used to treat psoriasis, which may increase AIS incidence in this population. Thus, psoriasis is increasingly acknowledged as an emerging risk factor for AIS alongside other systemic inflammatory diseases. Nevertheless, longitudinal studies are warranted to establish causal relationships between these conditions. As foregoing studies based on observational and cross-sectional data, can overestimate the strength of the association between psoriasis and AIS. Finally, studies reporting positive or statistically significant links are more susceptible to be published than those with negative results.
